# Treatment patterns, healthcare resource utilization, and costs among patients with idiopathic pulmonary fibrosis treated with antifibrotic medications in US-based commercial and Medicare Supplemental claims databases: a retrospective cohort study

**DOI:** 10.1186/s12890-020-01224-5

**Published:** 2020-07-11

**Authors:** Mitra Corral, Kathryn DeYoung, Amanda M. Kong

**Affiliations:** 1 Genentech, Inc., 1 DNA Way, South San Francisco, CA 94080 USA; 2 IBM Watson Health, 75 Binney Street, Cambridge, MA 02142 USA

**Keywords:** Adherence, Healthcare costs, Persistence, Respiratory

## Abstract

**Background:**

Pirfenidone and nintedanib are antifibrotic therapies which slow disease progression in idiopathic pulmonary fibrosis (IPF), an irreversible, progressive lung disease with poor prognosis. We compared adherence, persistence, and healthcare costs between patients initiating one of the two therapies.

**Methods:**

We used the IBM Watson Health Commercial and Medicare Supplemental claims databases to select patients with IPF with ≥1 pharmacy claim for pirfenidone or nintedanib between 10/1/2014 and 6/30/2018. Adherence (proportion of days covered ≥0.80) and persistence (time to a gap of ≥60 days without medication or switch to the other antifibrotic medication) based on the days’ supply and service date fields on claims were measured over a variable-length follow-up period. Healthcare costs, all-cause and respiratory-related, were measured over the persistent period and a fixed 12-month follow-up period. Inverse probability of treatment weights were applied to models comparing adherence, persistence, and costs between the two cohorts.

**Results:**

Overall, 799 pirfenidone patients and 656 nintedanib patients were identified. Similar proportions of patients were adherent in both cohorts (pirfenidone = 49% vs. nintedanib = 51%) and there was no significant difference in the odds of being adherent after weighting (odds ratio = 1.1, *p* = 0.513). The proportions of patients who discontinued/switched were also similar (pirfenidone = 41% vs. nintedanib 43%); however, in a weighted model, the hazards of discontinuation/switching was lower for the pirfenidone cohort (hazard ratio = 0.8, *p* = 0.032). While patients were persistent on therapy, weighted all-cause healthcare costs were comparable (pirfenidone = $11,272 vs. nintedanib = $11,987 per-patient per-month; *p* = 0.115), but weighted respiratory-related costs were significantly lower for the pirfenidone cohort ($9015 vs. $10,167 per-patient per-month, *p <* 0.001). Weighted annual total all-cause and respiratory-related healthcare costs were comparable between cohorts over the fixed 12-month follow-up period, but the pirfenidone cohort had significantly lower weighted annual mean antifibrotic drug costs than the nintedanib cohort ($68,850 vs. $77,033, *p* = 0.007).

**Conclusions:**

Pirfenidone use was associated with longer time to discontinuation/switch, lower antifibrotic drug costs, and lower respiratory-related total costs compared to nintedanib use.

## Background

Idiopathic pulmonary fibrosis (IPF) is an irreversible, progressive, fibrotic lung disease, in which the lung tissue becomes thick and scarred, reducing oxygenation of the blood and resulting in end organ damage [1, 2]. In the United States (US), IPF has an estimated annual incidence between 5.8 and 16.3 per 100,000 people per year and a prevalence of 13 to 20 cases per 100,000 [3–5]. The majority of patients are male and the risk of IPF for someone age 75 years is 8 times that of someone aged 45 to 54 years [6, 7]. Without treatment, the median survival in patients with IPF is 2–4 years after diagnosis [7, 8].

In 2014, the US Food and Drug Administration approved the first two antifibrotic therapies for IPF pirfenidone and nintedanib [9, 10]. In clinical trials, both antifibrotic therapies slowed disease progression as measured by a reduction in the decline in forced vital capacity compared to placebo [11–13]. Although the trials were individually underpowered for assessment of overall survival, analysis suggests treatment with either drug will improve overall survival compared to best supportive care [14–16].

Analyses of data on US Medicare beneficiaries have estimated that the annual cost of IPF to the US healthcare system, excluding medication costs, is $2 billion [17]. In particular, respiratory-related hospitalizations are associated with an elevated risk of mortality and a significant cost burden [18–21] There is limited real-world data on the impact of pirfenidone and nintedanib on healthcare resource utilization (HRU) and costs among patients with IPF. This retrospective cohort study compared treatment patterns, HRU, and direct healthcare costs between patients with IPF insured through commercial or Medicare Supplemental plans who were treated with pirfenidone or nintedanib.

## Methods

### Study design and data source

This observational retrospective cohort analysis utilized de-identified US administrative claims data covering October 1, 2014, to June 30, 2018, from three IBM MarketScan Databases: the Commercial Claims and Encounters (Commercial), the Medicare Supplemental and Coordination of Benefits (Medicare), and the Early View Databases [22]. The commercial database includes healthcare data of employees and their dependents covered under a variety of fee-for-service and managed care health plans. The Medicare database contains the healthcare experience of retirees with Medicare supplemental insurance paid for by employers. The Commercial and Medicare databases provided data access for the period of October 1, 2014, through September 30, 2017. Each database captures the inpatient medical, outpatient medical, and outpatient prescription drug data for its respective covered population, and together form a nationally representative sample of insured individuals living in the US.

The Early View database includes the same components as the Commercial and Medicare databases for the period of October 1, 2017, through June 30, 2018. Claims are available in the Early View database within 45 days of the end of the service month, and previous analysis has shown that over 97% of drug claims are fully adjudicated within the 30 days of the service date making it appropriate to use for treatment pattern analysis but not for HRU and cost analyses.

Study variables were based on enrollment information, International Classification of Diseases, 9th and 10th Revision, Clinical Modification (ICD-9-CM and ICD-10-CM) codes, Current Procedural Terminology 4th edition codes, Healthcare Common Procedure Coding System codes, and National Drug Codes. All study data were accessed with protocols compliant with US patient confidentiality requirements, including the Health Insurance Portability and Accountability Act of 1996 regulations (HIPAA). The databases used in the study are fully de-identified and compliant with the HIPAA. Research using the MarketScan Research Databases falls under Title 45 of the Code of Federal Regulations Part 46.101(b)(4) exemption from Institutional Review Board review because the databases contain only information that cannot be used to identify study subjects. As the data were de-identified, consent from patients was not sought.

### Patient selection and cohort assignment

The study population consisted of patients with at least one outpatient pharmacy claim for pirfenidone or nintedanib from October 1, 2014, to June 30, 2018. The first such claim during the study period was set as the index date. Patients were required to be at least 40 years old on the index date, have continuous enrollment with medical and prescription coverage for at least 12 months before the index date (baseline period), and have at least one non-diagnostic inpatient or outpatient claim with an diagnosis code for IPF (ICD-9-CM: 516.3 and 516.31; ICD-10-CM: J84.112) between the baseline period and June 30, 2018. Patients were excluded if they had evidence of other interstitial lung diseases (hypersensitivity pneumonitis, diffuse connective tissue disease, rheumatoid arthritis and other inflammatory polyarthropathies, radiation fibrosis, pneumoconiosis, asbestosis, silicosis or talcosis, berylliosis and other inorganic dusts, unspecified pneumoconiosis, sarcoidosis) between the index date and June 30, 2018.

### Dataset development

Three datasets were constructed from the set of patients who met the above criteria: the treatment patterns dataset, the variable-length HRU and cost while persistent dataset, and the 12-month fixed-length HRU and cost dataset. Patients in each dataset were stratified into two cohorts based on the antifibrotic received on the index date: pirfenidone or nintedanib.

The treatment patterns dataset contained all eligible patients and followed them from 12 months before their index date until the earliest of the following: inpatient death, end of continuous enrollment, or June 30, 2018 (end of the Early View data). The variable-length HRU and cost while persistent dataset comprised patients who had an index date on or before September 30, 2017 (end of non-Early View data). Patients were followed from 12 months before their index date until the earliest of the following: inpatient death, end of continuous enrollment, treatment discontinuation (defined below), or September 30, 2017. The 12-month fixed-length HRU and cost dataset comprised patients who had an index date on or before September 30, 2016 and at least 12 months of continuous enrollment in non-Early View data. Patients were followed from 12 months before their index date through 12 months after their index date, regardless of the length of time they persisted on their index medication.

### Baseline characteristics

Demographic characteristics, including age, sex, and geographic region, were measured on the index date. Baseline clinical characteristics were measured during the baseline period and included: the Deyo-Charlson Comorbidity Index (CCI) [23], the CCI excluding codes related to chronic obstructive pulmonary disease (COPD), supplemental oxygen use, opioid prescriptions, and claims with diagnosis codes for cardiovascular disease (including stroke specifically), COPD, cancer (including lung cancer specifically), dyspnea, and recent pneumonia (3 months preceding the index date). Patients who were treated at an Interstitial Lung Disease (ILD) Center of Excellence as identified by the zip code of the facility were also flagged. Centers of Excellence were defined as medical centers with specific expertise in the treatment of pulmonary fibrosis, as recognized by the Pulmonary Fibrosis Foundation.

#### Outcomes

##### Treatment patterns

Treatment adherence, discontinuation, persistence, switching, and re-initiation were assessed during the variable follow-up period for all patients in the treatment patterns dataset. Medication adherence was measured by the proportion of days covered (PDC) defined as the sum of days’ supply during the follow-up period divided by the length of the follow-up. To account for titration, an additional 7 days were added to the days’ supply of the first pirfenidone prescription claim unless the supply was 207 pills for 30 days, which would be correct if following the recommended titration schedule. Overlapping days’ supply between consecutive fills were appended, and the maximum PDC was truncated at a value of 1.0. Patients with a PDC greater than 0.8 were considered adherent.

Discontinuation was defined as having a gap in treatment of ≥60 days. The date of the last day supply before the first 60-day gap was the discontinuation date. Patients who had a lung transplant before the end of a 60-day gap in treatment were not considered to have discontinued. Switching was defined as a filled prescription for the alternate antifibrotic medication. The date of the new prescription was defined as the switching date. The persistence period was defined as the time from the index date to the earliest of the following: discontinuation, lung transplantation, switching, or end of follow-up. Re-initiation was defined as having a new claim for the index drug after discontinuation.

##### HRU & Costs

HRU and cost outcomes were: inpatient admissions (excluding lung transplants), outpatient services (including emergency department visits and physician office visits), and outpatient pharmacy claims. Because of variable follow-up, per-patient per-month (PPPM) all-cause, respiratory-related, and index drug-related HRU and costs were recorded for the period of persistence on index medication for all patients in the variable-length HRU and cost dataset. Per-patient per-year (PPPY; i.e., annual) all-cause, respiratory-related, and index drug-related HRU and costs were recorded for the 12-month post-index period for all patients in the fixed-length HRU and cost dataset. All-cause utilization included all claims, regardless of diagnosis, procedure, or medication. Respiratory-related utilization included inpatient claims with a primary diagnosis of respiratory disease, outpatient claims with a diagnosis of a respiratory disease in the first position and pharmacy claims for respiratory therapies (antibiotics for pneumonia or acute respiratory infection, antifibrotic medications, oral corticosteroids, azathioprine, N-acetylcysteine and mycophenolate mofetil).

Costs were the paid amounts of fully adjudicated claims, including insurer and health plan payments, as well as patient cost-sharing in the form of copayment, deductible, and coinsurance. All-cause costs include the costs on all claims; while respiratory-related costs include the cost of claims determined to be respiratory-related as defined above. All costs were inflated to 2017 US dollars using the medical care component of the Consumer Price Index [24].

##### Statistical analysis

Frequencies and percentages were reported for categorical variables, and statistical significance between treatment groups was determined using Chi-square tests. Mean and standard deviation (SD) were reported for continuous variables, and statistical significance between treatment groups was determined using two-sample *t*-tests.

Inverse probability of treatment weighting (IPTW) was used to address cohort imbalances via a logistic regression model, treating pirfenidone vs. nintedanib as the dependent variable. Covariates included age group (40–44, 45–54, 55–64, 65–74,75–79, 80+), sex, geographic region of residence, CCI without COPD codes, evidence of COPD, evidence of stroke, evidence of recent pneumonia, and baseline visit to an ILD Center of Excellence. Cohort balances were evaluated by standardized mean difference where a value of less than or equal to 0.1 indicates a good balance (Additional file 1).

HRU results are unweighted and therefore, do not account for differences in patient characteristics. Non-PPPM measures are unadjusted for differences in follow-up time during persistent period. Logistic regression was used to estimate the odds of being adherent to treatment based on a PDC greater than or equal to 0.8. Cox proportional hazard regression models with accompanying Kaplan-Meier curves were employed to estimate the time to discontinuation or switching among all patients and time to discontinuation among patients who did not switch medications. All-cause, respiratory-related, and index drug-related costs for both the period of persistence and the 12-month follow-up cohort were modeled using a generalized linear models with a log link and gamma distribution. The main explanatory variables in all models was index antifibrotic treatment. The same covariates in the IPTW model were included in all multivariable models and all models were weighted by IPTW. Odds ratios (OR) and hazard ratios (HR) were reported with 95% confidence intervals (CI). Predicted costs were estimated for each group using the recycled prediction method and bootstrap resampling to estimate 95% CI. The alpha level for all statistical tests was 0.05.

Statistical analyses were conducted using SAS 9.4 and R.

## Results

### Patient characteristics

**Fig. 1:**
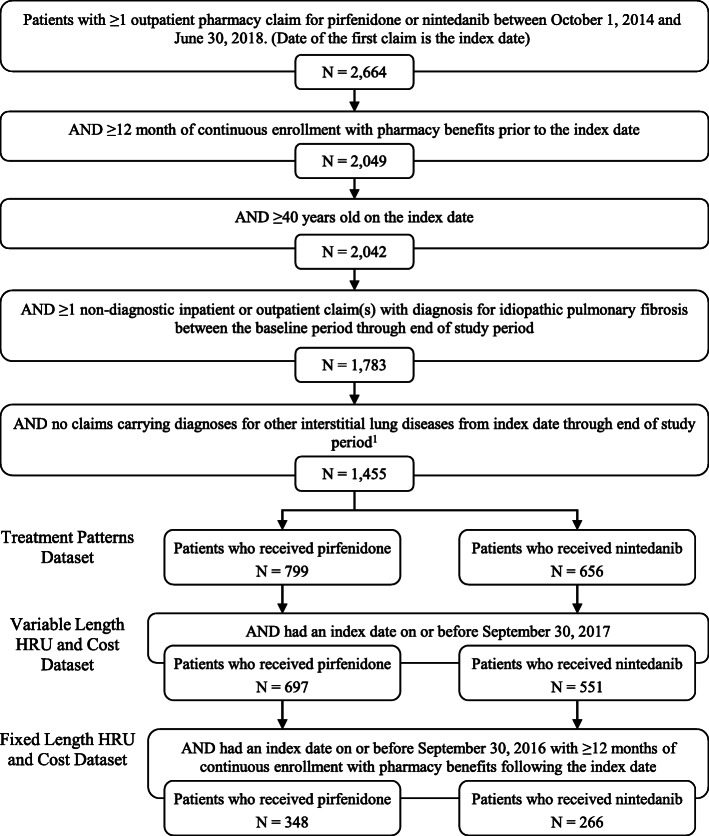
Patient selection ^1^Other interstitial lung diseases are defined as diagnoses of hypersensitivity pneumonitis, diffuse connective tissue disease, rheumatoid arthritis and other inflammatory polyarthropathies, radiation fibrosis, pneumoconiosis, asbestosis, silicosis or talcosis, berylliosis and other inorganic dusts, unspecified pneumoconiosis, and sarcoidosis. HRU, healthcare resource utilization

Overall, 1455 patients with IPF met the selection criteria (Fig. 1). Of these, 799 patients received pirfenidone, and 656 received nintedanib. The mean patient age at index was 70.9 years, and 31.3% of patients were female (Table 1). Baseline characteristics were generally similar between the two cohorts; however, there were some differences in regional distribution and fewer pirfenidone users had a baseline diagnosis of COPD than nintedanib users (44.9% vs. 50.8%, *P* = 0.027).

**Table 1:** Baseline patient characteristics

	All Patients	Pirfenidone	Nintedanib	*P* Value^1^
	***N*** = 1455	***N*** = 799	***N*** = 656	
**Age, mean (SD)**	70.9 (9.5)	70.6 (9.4)	71.3 (9.7)	0.222
**Age categories, years, n (%)**				0.581
40–44	1 (0.1)	1 (0.1)	(0.0)	
45–54	68 (4.7)	36 (4.5)	32 (4.9)	
55–64	377 (25.9)	212 (26.5)	165 (25.2)	
65–74	445 (30.6)	255 (31.9)	190 (29.0)	
75–79	269 (18.5)	140 (17.5)	129 (19.7)	
≥ 80	295 (20.3)	155 (19.4)	140 (21.3)	
**Female, n (%)**	456 (31.3)	256 (32.0)	200 (30.5)	0.525
**Region, n (%)**				0.010
Northeast	271 (18.6)	157 (19.6)	114 (17.4)	
North Central	457 (31.4)	236 (29.5)	221 (33.7)	
South	554 (38.1)	294 (36.8)	260 (39.6)	
West	169 (11.6)	111 (13.9)	58 (8.8)	
Unknown	4 (0.3)	1 (0.1)	3 (0.5)	
**CCI, mean (SD)**	2.1 (2.0)	2.1 (2.0)	2.1 (1.9)	0.512
** excluding COPD, mean (SD)**	1.9 (2.0)	1.9 (2.0)	1.9 (1.9)	0.680
**Proxies of disease severity, n (%)**				
Presence of supplemental oxygen use	868 (59.7)	478 (59.8)	390 (59.5)	0.885
Diagnosis of dyspnea	1131 (77.7)	621 (77.7)	510 (77.7)	0.992
Prescription for ≥1 opioid(s)	557 (38.3)	306 (38.3)	251 (38.3)	0.989
**Presence of comorbid conditions, n (%)**				
Any cardiovascular disease	640 (44.0)	354 (44.3)	286 (43.6)	0.787
Stroke	37 (2.5)	23 (2.9)	14 (2.1)	0.369
COPD	692 (47.6)	359 (44.9)	333 (50.8)	0.027
Emphysema	189 (13.0)	99 (12.4)	90 (13.7)	0.453
Bronchitis	101 (6.9)	51 (6.4)	50 (7.6)	0.355
Chronic bronchitis	156 (10.7)	73 (9.1)	83 (12.7)	0.031
Chronic airway obstruction	562 (38.6)	289 (36.2)	273 (41.6)	0.034
Cancer	269 (18.5)	154 (19.3)	115 (17.5)	0.394
Lung cancer	31 (2.1)	21 (2.6)	10 (1.5)	0.147
Recent pnemonia^2^	149 (10.2)	77 (9.6)	72 (11.0)	0.402
**All-cause total costs, mean (SD)**	$31,933 ($54,125)	$31,849 ($44,706)	$32,035 ($63,781)	0.948
**Visit to ILD center of excellence**^**3**^**, n (%)**	110 (7.6)	57 (7.1)	53 (8.1)	0.497

*CCI* Charlson Comorbidity Index, *COPD* chronic obstructive pulmonary disease, *SD* standard deviation

^1^Pirfenidone vs. nintedanib

^2^Pneumonia diagnosis in the 3 months immediately preceding the index date

^3^ Patient was treated at an Interstitial Lung Disease (ILD) Center of Excellence as identified by zip code of facility. Centers of excellence were defined as medical centers with specific expertise in the treatment of pulmonary fibrosis, as recognized by the Pulmonary Fibrosis Foundation

### Treatment patterns

The mean length of follow-up data available for treatment pattern analysis was 480.2 days (median: 403 days) among pirfenidone-treated patients and 446.6 days (median: 383 days) among nintedanib-treated patients (*P* = 0.064). Descriptively, the mean (SD) PDC in all patients and in both treatment groups was 0.66 (0.35) (Table 2). Over the variable-length follow-up, 48.8 and 50.5% of patients were considered adherent in the pirfenidone and nintedanib cohorts, respectively (*P* = 0.532).

**Table 2:** Unadjusted treatment patterns

	All Patients	Pirfenidone	Nintedanib	*P* Value^1^
	***N*** = 1455	***N*** = 799	***N*** = 656	
**Follow-Up**				
Days of follow-up after index date, mean (SD)	465.0 (343.9)	480.2 (351.4)	446.6 (334.0)	0.064
**Adherence**				
Total days with index drug, mean (SD)^2^	269.0 (277.8)	282.7 (289.7)	252.4 (261.8)	0.038
PDC during follow-up, mean (SD)	0.66 (0.35)	0.66 (0.35)	0.66 (0.35)	0.910
PDC ≥ 0.80, n (%)	721 (49.6)	390 (48.8)	331 (50.5)	0.532
**Persistence**				
Duration of persistence in days, mean (SD)^3^	286.8 (301.4)	305.2 (314.7)	264.4 (282.9)	0.010
Discontinuation or switching, n (%)^3^	610 (41.9)	326 (40.8)	284 (43.3)	0.338
Days on index medication in patients who discontinued or switched, mean (SD)	167.4 (189.8)	171.2 (190.5)	163.1 (189.4)	0.598
Switching, n (%)	144 (9.9)	86 (10.8)	58 (8.8)	0.222
Days on index medication in patients who switched, mean (SD)	297.9 (210.1)	251.4 (184.7)	366.9 (227.5)	0.001
**Other discontinuation-related outcomes**				
Lung transplant, n (%)	82 (5.6)	53 (6.6)	29 (4.4)	0.069
Discontinuation before lung transplant, n (%)^3,4^	36 (43.9)	21 (39.6)	15 (51.7)	0.291
Re-initiation of index medication, n (%)^5^	101 (16.1)	61 (17.8)	40 (14.0)	0.197
Days to re-initiation, mean (SD)^6^	157.3 (122.0)	150.4 (107.4)	167.9 (142.2)	0.484

*PDC* proportion of days covered, *SD* standard deviation

^1^ Pirfenidone vs. nintedanib

^2^ To account for titration, an additional 7 days were added to the day supply of the first pirfenidone prescription claim, unless the supply was 207 pills for 30 days, which is correct if following the recommended titration schedule

^3^ Patients with a lung transplant before the end of a gap of ≥60 days were not considered to have discontinued

^4^ Among those with a lung transplant

^5^ Among those who discontinued treatment

^6^The minimum time to re-initiation is 61 days (as discontinued is defined as a gap if 60 days or more)

The mean (SD) duration of persistence with index medication was significantly longer in the pirfenidone vs. nintedanib cohort in an comparison unadjusted for differences in patient characteristics (305.2 [314.7] vs. 264.4 [282.9] days; *P* = 0.010) (Table 2). Approximately 40% of patients in both cohorts discontinued their index medication (40.8% vs. 43.3%, *P* = 0.338). Among those with discontinued, 17.8% of patients re-initiated pirfenidone, and 14.0% re-initiated nintedanib (*P* = 0.197). The mean (SD) time to re-initiation of index medication was not significantly different between pirfenidone-treated and nintedanib-treated patients (150.4 [107.4] vs. 167.9 [142.2] days; *P* = 0.484). Among the 10.8% of pirfenidone patients and 8.8% of nintedanib patients who switched to the alternate antifibrotic, the time between the index and switching date was shorter for pirfenidone patients (251.4 [184.7] vs. 366.9 [227.5] days; *P* = 0.001).

**Fig. 2:**
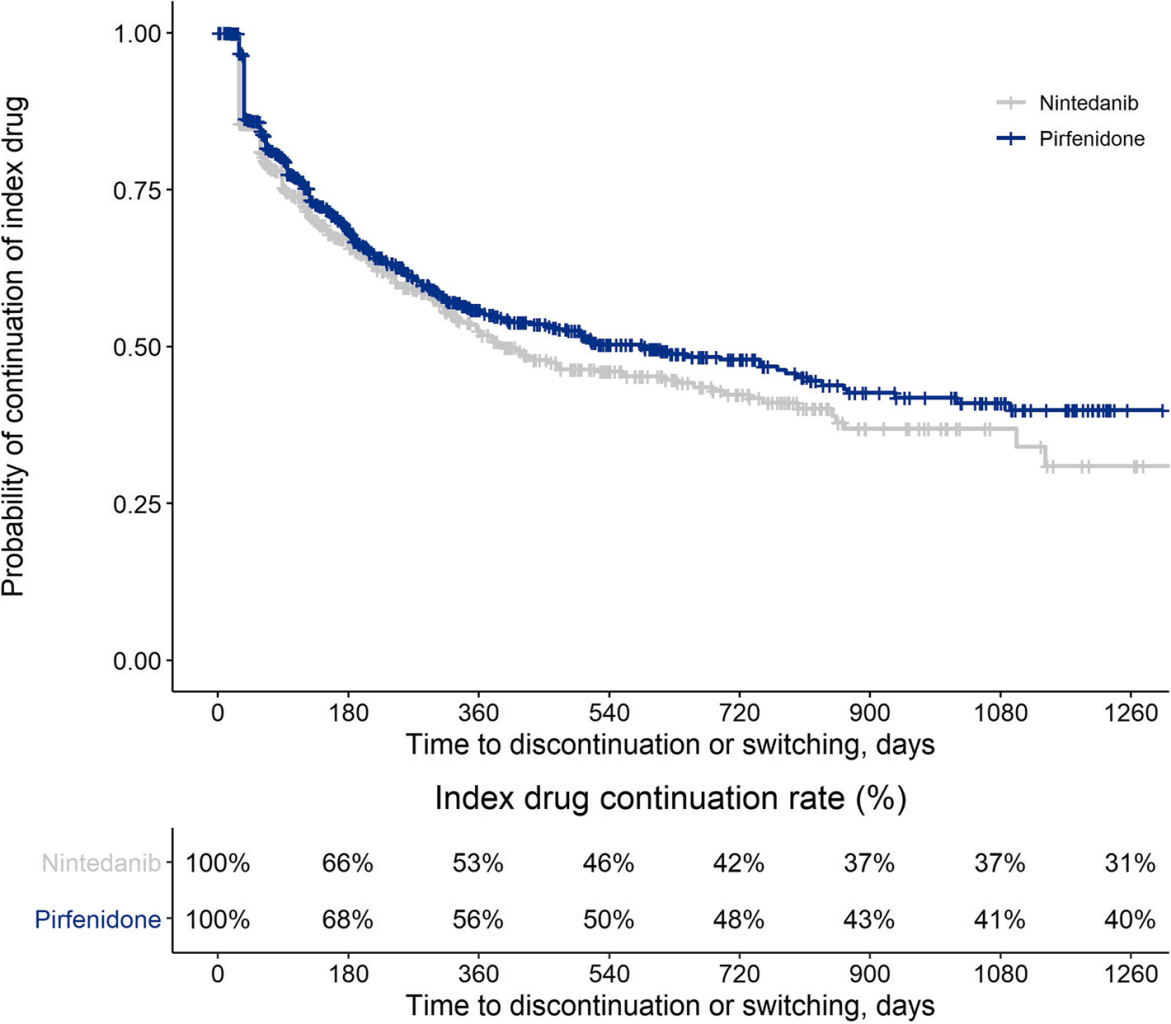
Kaplan-Meier plot of time to discontinuation/switching among patients with IPF treated with pirfenidone or nintedanib

In models weighted to account for differences in baseline characteristics, no significant difference in odds of adherence (OR [95% CI]) was observed between patients treated with pirfenidone and patients treated with nintedanib (1.07 [0.88–1.30]; *P* = 0.513). However, indexing on pirfenidone was associated with a longer time to discontinuation or switching than indexing on nintedanib (HR [95% CI]: 0.84 [0.71–0.99], *P* = 0.032) (Fig. 2). This association was stronger in the subgroup of patients who did not switch therapies (*n* = 1311; HR [95% CI]: 0.75 [0.63–0.91], *P* = 0.003).

### HRU and costs while persistent on index antifibrotic

Among the 697 pirfenidone users and 551 nintedanib users who indexed before September 30, 2017, the mean length of follow-up while persistent was 260.7 days (median, 179 days) in the pirfenidone cohort and 229.6 days (median, 134 days) in the nintedanib cohort (*P* = 0.025). While persistent with antifibrotic therapy, a significantly greater proportion of patients treated with pirfenidone than patients treated with nintedanib had at least one all-cause inpatient hospitalization (24.7% vs. 18.0%; *P* = 0.004) or respiratory-related inpatient hospitalization (13.9% vs. 8.9%; *P* = 0.006) (Table 3). The differences between pirfenidone and nintedanib cohorts in the number of all-cause inpatient hospitalizations (0.06 [0.18] vs. 0.05 [0.16]; *P* = 0.208) and respiratory-related inpatient hospitalizations (0.03 [0.14] vs. 0.02 [0.11]; *P* = 0.166) during the persistent period were not statistically significant.

**Table 3:** Post-index healthcare utilization while persistent on antifibrotics

	All Patients	Pirfenidone	Nintedanib	*P* Value^1^
	***N*** = 1248	***N*** = 697	***N*** = 551	
**Follow-Up**				
Days of follow-up after index date, mean (SD)	247.0 (243.7)	260.7 (251.1)	229.6 (233.0)	0.025
**All-cause utilization**				
Inpatient hospitalization^2^, n (%)	271 (21.7)	172 (24.7)	99 (18.0)	0.004
Inpatient hospitalizations PPPM, mean (SD)	0.06 (0.17)	0.06 (0.18)	0.05 (0.16)	0.208
Length of stay, mean (SD), days	5.0 (4.1)	4.8 (3.4)	5.4 (5.1)	0.239
Median	4	4	4	
Any outpatient visit, n (%)	1195 (95.8)	666 (95.6)	529 (96.0)	0.692
Emergency department visit	308 (24.7)	174 (25.0)	134 (24.3)	0.793
Visits PPPM, mean (SD)	0.07 (0.23)	0.07 (0.20)	0.08 (0.26)	0.163
Physician office visit	1092 (87.5)	613 (87.9)	479 (86.9)	0.590
Visits PPPM, mean (SD)	1.2 (1.0)	1.2 (1.0)	1.2 (1.0)	0.724
Pharmacy, n (%)	1248 (100.0)	697 (100.0)	551 (100.0)	1.000
Medication claims PPPM, mean (SD)	4.3 (2.6)	4.2 (2.5)	4.3 (2.7)	0.335
**Respiratory-related utilization**				
Inpatient hospitalization^2^, n (%)	146 (11.7)	97 (13.9)	49 (8.9)	0.006
Inpatient hospitalizations PPPM, mean (SD)	0.03 (0.13)	0.03 (0.14)	0.02 (0.11)	0.166
Length of stay, mean (SD), days	5.3 (5.4)	4.6 (3.2)	6.5 (8.1)	0.052
Median	4	4	4	
Any outpatient visit, n (%)	1147 (91.9)	640 (91.8)	507 (92.0)	0.901
Emergency department visit	173 (13.9)	101 (14.5)	72 (13.1)	0.470
Visits PPPM, mean (SD)	0.04 (0.14)	0.03 (0.13)	0.04 (0.16)	0.196
Physician office visit	1025 (82.1)	577 (82.8)	448 (81.3)	0.499
Visits PPPM, mean (SD)	0.68 (0.63)	0.68 (0.65)	0.67 (0.60)	0.913
Pharmacy, n (%)	1248 (100.0)	697 (100.0)	551 (100.0)	1.000
Medication claims PPPM, mean (SD)	1.4 (0.7)	1.4 (0.7)	1.5 (0.8)	0.026

*PPPM* per-person per-month, *SD* standard deviation

^1^ Pirfenidone vs. nintedanib

^2^ Excluding inpatient hospitalizations for a lung transplant

**Fig. 3:**
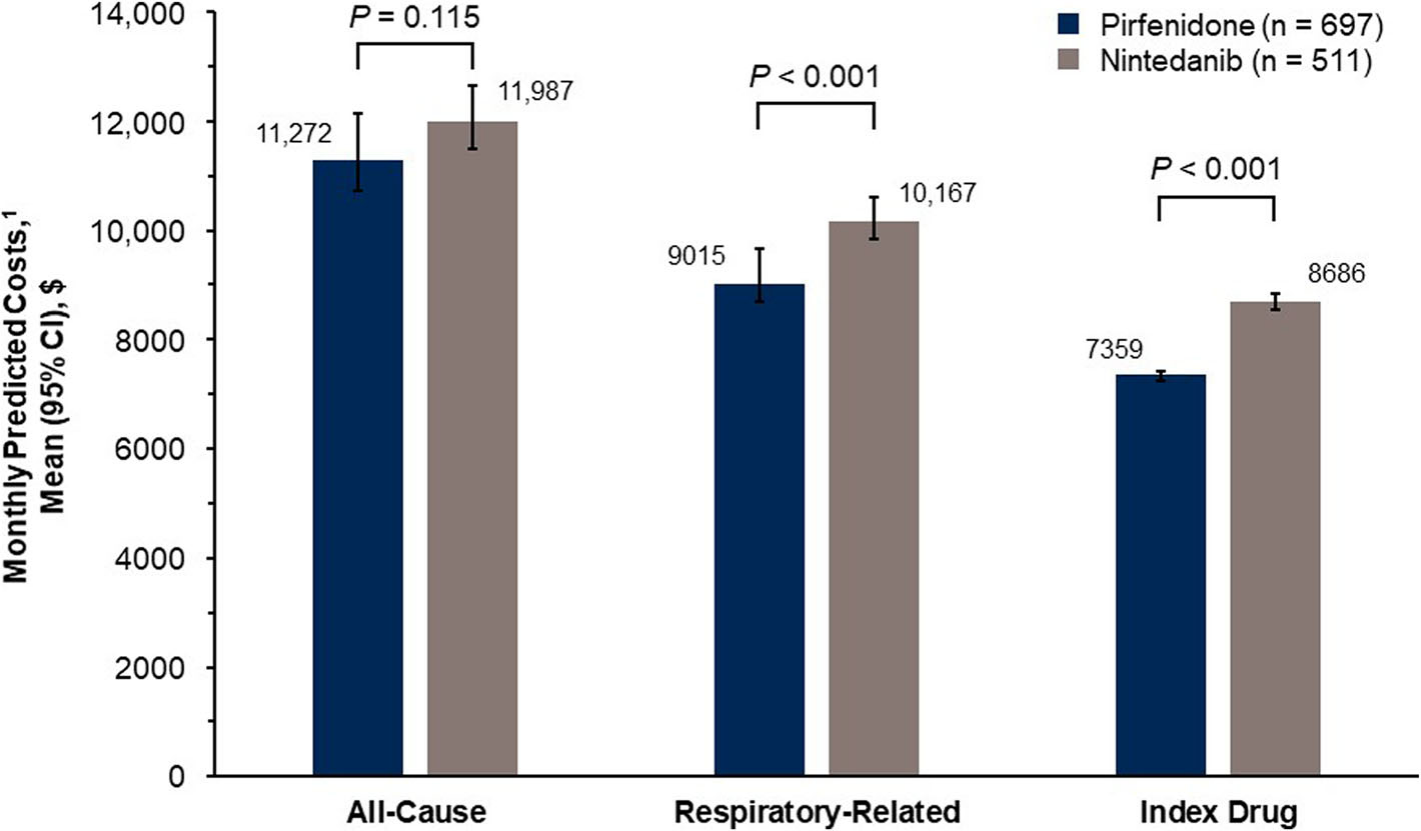
Adjusted healthcare costs per-person per-month while persistent on antifibrotics. ^1^Predicted costs were estimated using the recycled prediction method and bootstrap resampling to estimate 95% CIs

While patients were persistent with therapy, the predicted all-cause healthcare costs based on the weighted multivariable models were comparable between pirfenidone and nintedanib treated patients ($11,272 vs. $11,987 PPPM, *P* = 0.115), but predicted respiratory-related costs were significantly lower in patients treated with pirfenidone than patients treated with nintedanib ($9015 vs. $10,167 PPPM, *P* < 0.001) (Fig. 3). While patients were persistent with therapy, predicted drug costs were significantly lower in patients treated with pirfenidone than patients treated with nintedanib ($7359 vs. $8686 PPPM; *P* < 0.001).

### HRU and costs during the 12-month fixed length follow-up

Among the 348 pirfenidone users and 266 nintedanib users with at least 12 months of post-index non-Early View data, 12-month all-cause and respiratory-related healthcare utilization rates were generally similar between cohorts (Table 4). Among patients treated with pirfenidone or nintedanib, roughly one-quarter had at least one all-cause inpatient hospitalization (26.1% vs. 24.4%; *P* = 0.629). While there was no statistically significant difference in the proportions of patients with at least one respiratory-related inpatient hospitalization, the mean (SD) length of stay for respiratory-related hospitalizations was significantly shorter in patients treated with pirfenidone than patients treated with nintedanib (4.1 [2.9] vs. 6.3 [5.4] days; *P* = 0.033).

**Table 4:** Post-index healthcare utilization among patients with 12 months of continuous enrollment post-index

	All Patients	Pirfenidone	Nintedanib	*P* Value^1^
	***N*** = 614	***N*** = 348	***N*** = 266	
**All-cause utilization**				
Inpatient hospitalization^2^, n (%)	156 (25.4)	91 (26.1)	65 (24.4)	0.629
Length of stay, mean (SD), days	4.4 (3.7)	4.2 (3.5)	4.8 (4.1)	0.263
Median	3	3	3.5	
Any outpatient visit, n (%)	610 (99.1)	346 (99.4)	264 (99.2)	1.000
Emergency department visit	206 (33.6)	112 (32.2)	94 (35.3)	0.412
Physician office visit	600 (97.7)	340 (97.7)	260 (97.7)	0.972
Pharmacy, n (%)	614 (100.0)	348 (100.0)	266 (100.0)	1.000
Medication claims PPPY, mean (SD)	49.6 (27.0)	49.7 (26.4)	49.5 (27.8)	0.938
**Respiratory-related utilization**				
Inpatient hospitalization^2^, n (%)	70 (11.4)	45 (12.9)	25 (9.4)	0.172
Length of stay, mean (SD), days	4.9 (4.1)	4.1 (2.9)	6.3 (5.4)	0.033
Median	4	3.5	4	
Any outpatient visit, n (%)	607 (98.9)	343 (98.6)	264 (99.2)	0.705
Emergency department visit	116 (18.9)	64 (18.4)	52 (19.5)	0.716
Physician office visit	597 (97.2)	338 (97.1)	259 (97.4)	0.856
Pharmacy, n (%)	614 (100.0)	348 (100.0)	266 (100.0)	1.000
Medication claims PPPY, mean (SD)	14.6 (8.0)	14.4 (7.5)	14.8 (8.6)	0.517

*PPPY* per-person per-year, *SD* standard deviation

^1^ Pirfenidone vs. nintedanib

^2^ Excluding inpatient hospitalizations for a lung transplant

**Fig. 4:**
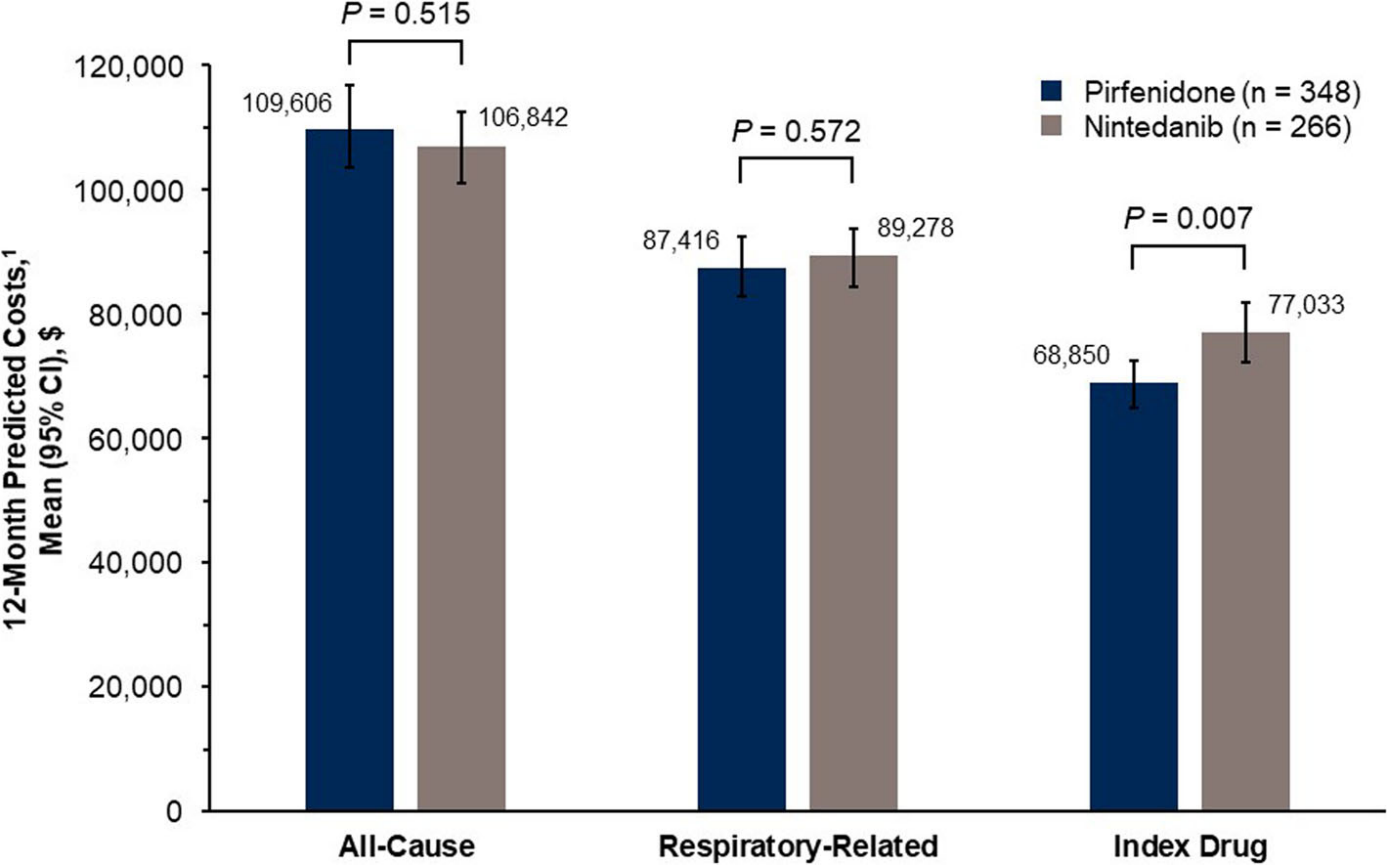
Adjusted healthcare costs per-person per-year among patients with 12 months of continuous enrollment post-index. ^1^Predicted costs were estimated using the recycled prediction method and bootstrap resampling to estimate 95% CIs

In multivariable models, the annual total all-cause and respiratory-related healthcare costs among the subset of patients with 12 months of post-index enrollment were comparable between patients treated with pirfenidone and patients treated with nintedanib (Fig. 4). However, patients treated with pirfenidone had significantly lower adjusted annual mean drug costs than those treated with nintedanib ($68,850 vs. $77,033 PPPY, *P* = 0.007).

## Discussion

In a real-world setting, patients with IPF who were treated with pirfenidone had a longer time to discontinuation or switching and similar odds of adherence compared to patients treated with nintedanib. While persistent on index medication, patients treated with pirfenidone had 15% lower index drug costs, 11% lower respiratory-related costs, and similar all-cause costs than patients treated with nintedanib. This was in spite of higher utilization of all-cause and respiratory-related inpatient services while they were persistent on their index medication. Among the subset of patients with 12 months of follow-up data, all-cause and respiratory-related adjusted annual costs were similar regardless of index medication; however, index drug-related costs were lower for patients who indexed on pirfenidone.

Adherence to and persistence with antifibrotic therapies in IPF are important for maintaining treatment benefits and slowing lung function decline; however, there is limited existing data on treatment patterns as pirfenidone and nintedanib have only been recently approved to treat IPF in the US [25–27]. Two recent studies of US Medicare and commercially-insured patients found that those who indexed on pirfenidone had higher adherence and longer persistence than patients who indexed on nintedanib; however, the mean follow-up time was less than half of that reported in this study and there are difference in the patient populations and patient selection criteria [25, 26]. In an analysis of Swedish patients with IPF treated with pirfenidone, 24.7–41.8% (depending on data source) were persistent at 1 year compared with over 50% in our study; however, they used a 30-day gap to define discontinuation compared to the 60-day gap used in this study [27]. Treatment pattern analysis was not available on patients who indexed on nintedanib in the Swedish analysis due to low utilization during the study time frame.

To our knowledge, no analyses comparing healthcare costs between IPF patients treated with pirfenidone and IPF patients treated with nintedanib have been published. Prior to the approval of pirfenidone and nintedanib, analyses of Medicare and commercially insured patients with IPF have estimated mean annual healthcare costs ranging from $26,000 to $ 59,000 and mean annual medical costs (total costs minus pharmacy costs) ranging from $20,000 to $52,000 [17, 20, 28]. In our analysis, index medication costs comprised a large proportion of total costs. When index medication costs were excluded, mean adjusted annual costs for patients treated with pirfenidone and nintedanib were $40,756 and $29,809 respectively, which are within the ranges of the previous estimates of mean healthcare costs of IPF prior to the introduction of antifibrotic therapies.

A previous analysis has shown that acute exacerbations in the first 6 months after diagnosis of IPF and a greater decline in forced vital capacity are associated with increased healthcare utilization and shorter overall survival [21]. In studies of commercially insured patients with IPF prior to the approval of pirfenidone and nintedanib, over 37% of patients had at least one all-cause inpatient admission during a 1-year follow-up period [20, 29]. Treatment with antifibrotics has been demonstrated to reduce the decline in lung function with the assumption that this will reduce healthcare utilization and improve overall survival, but effective treatment requires that patients are adherent to and persistent on medication. Future real-world studies can examine whether the differences in medication persistence observed in this analysis persist as more data become available and translate to improved clinical and economic outcomes.

### Limitations

There are several limitations that should be considered when interpreting the findings of this study. First, as with any analysis of healthcare claims data, the adherence and persistence results reflect patterns in prescription fills (and not necessarily use) of the index medications; therefore, they may not fully capture usage patterns of antifibrotic therapy among patients with IPF. Second, unadjusted results for healthcare utilization did not account for potential differences in patient characteristics between treatment groups. Third, cost comparisons were adjusted for some potential confounders; however, residual confounding may exist due to the lack of clinical data included in administrative claims data or other unobserved differences between cohorts. Because patients were selected based on initiation of pirfenidone vs. nintedanib and clinical notes are not available, we were not able to control for years since initial diagnosis. Measures of disease severity, such as results of pulmonary function tests and GAP stage, were also not available. Fourth, only a small number of patients had 12 months of non-Early View follow-up data, so the analysis may be underpowered to detect differences in HRU and costs. Fifth, the proportions of patients with specific utilization types measured during the persistent period did not account for variable follow-up time. Sixth, prior to the approval of these medications there was no effective treatment for IPF; therefore, patients in this analysis may be sicker on average than future patients who have access to these medications upon initial diagnosis. Patients who did not survive 12 months after index medication initiation did not meet the continuous enrollment criteria for the fixed 12-month follow-up outcomes. Finally, analysis was restricted to patients with commercial or Medicare Supplemental insurance and findings may not extend to patients with other types of insurance or no insurance.

## Conclusions

After controlling for patient differences, IPF patients on pirfenidone remained on index therapy significantly longer than nintedanib patients. While patients were persistent on their index medication, weighted index drug costs and respiratory-related total costs were significantly lower among patients treated with pirfenidone compared to nintedanib patients.

## Supplementary information

**Additional file 1.** Demographic and clinical characteristics before and after weighting for patients with index date prior to 10/1/2017.

## Data Availability

The proprietary data that support the findings of this study are available from IBM Watson Health but restrictions apply to the availability of these data, and so they are not publicly available. Please contact AMK (akong@us.ibm.com) IBM Watson Health for information on licensing the MarketScan data.

## Abbreviations

CCI: Deyo-Charlson Comorbidity Index
CI: Confidence interval
COPD: Chronic obstructive pulmonary disease
HIPAA: Health Insurance Portability and Accountability Act
HR: Hazard ratio
HRU: Healthcare resource utilization
ICD-9-CM: International Classification of Diseases, 9th Revision, Clinical Modification
ICD-10-CM: International Classification of Diseases, 10th Revision, Clinical Modification
ILD: Interstitial lung disease
IPF: Idiopathic pulmonary fibrosis
IPTW: Inverse probability of treatment weighting
OR: Odds ratio
PDC: Proportion of days covered
PPPM: Per-patient per-month
PPPY: Per-patient per-year
SD: Standard deviation
US: United States
