# Supplementary material for: Treatment patterns, healthcare resource utilization, and costs among patients with idiopathic pulmonary fibrosis treated with antifibrotic medications in US-based commercial and Medicare Supplemental claims databases: a retrospective cohort study
Source: BMC Pulm Med. 2020 Jul 11;20:188. doi: 10.1186/s12890-020-01224-5 (PMC7353678; doi:10.1186/s12890-020-01224-5)
Supplement: Supplementary file 1 — Additional file 1. Demographic and clinical characteristics before and after weighting for patients with index date prior to 10/1/2017. [file 12890_2020_1224_MOESM1_ESM.docx]

Additional File 1. Demographic and clinical characteristics before and after weighting for patients with index date prior to 10/1/2017

| Variable | **Pirfenidone** | **Nintedanib** | **SMD^1^** | **Pirfenidone** | **Nintedanib** | **SMD^1^** |
| --- | --- | --- | --- | --- | --- | --- |
| n | 697 | 551 |  | 1269.2 | 1225.3 |  |
| Age (%) |  |  | 0.111 |  |  | 0.027 |
| 65-74 | 222 (31.9) | 156 (28.3) |  | 30.7 | 29.7 |  |
| 40-54 | 34 ( 4.9) | 28 ( 5.1) |  | 5.1 | 5.0 |  |
| 55-64 | 186 (26.7) | 137 (24.9) |  | 26.1 | 25.9 |  |
| 75-79 | 121 (17.4) | 108 (19.6) |  | 18.1 | 18.4 |  |
| 80+ | 134 (19.2) | 122 (22.1) |  | 20.0 | 20.9 |  |
| Sex = Female (%) | 220 (31.6) | 171 (31.0) | 0.011 | 30.9 | 32.4 | 0.032 |
| Region (%) |  |  | 0.154 |  |  | 0.041 |
| South/Unknown | 255 (36.6) | 223 (40.5) |  | 38.0 | 38.6 |  |
| Northeast | 141 (20.2) | 99 (18.0) |  | 19.2 | 19.0 |  |
| North Central | 205 (29.4) | 176 (31.9) |  | 31.3 | 29.8 |  |
| West | 96 (13.8) | 53 ( 9.6) |  | 11.5 | 12.6 |  |
| Deyo CCI excluding COPD (median [IQR]) | 1.0 [0.0, 3.0] | 1.0 [0.0, 3.0] | 0.049 | 1.0 [0.0, 3.0] | 1.0 [0.0, 3.0] | 0.024 |
| COPD = Yes (%) | 313 (44.9) | 281 (51.0) | 0.122 | 47.4 | 47.8 | 0.007 |
| Stroke = Yes (%) | 18 ( 2.6) | 11 ( 2.0) | 0.039 | 2.3 | 2.5 | 0.013 |
| Pneumonia^2^ = Yes (%) | 67 ( 9.6) | 58 (10.5) | 0.030 | 10.1 | 9.6 | 0.016 |
| ILD Center of Excellence^3^ = Yes (%) | 52 ( 7.5) | 46 ( 8.3) | 0.033 | 8.0 | 8.1 | 0.004 |


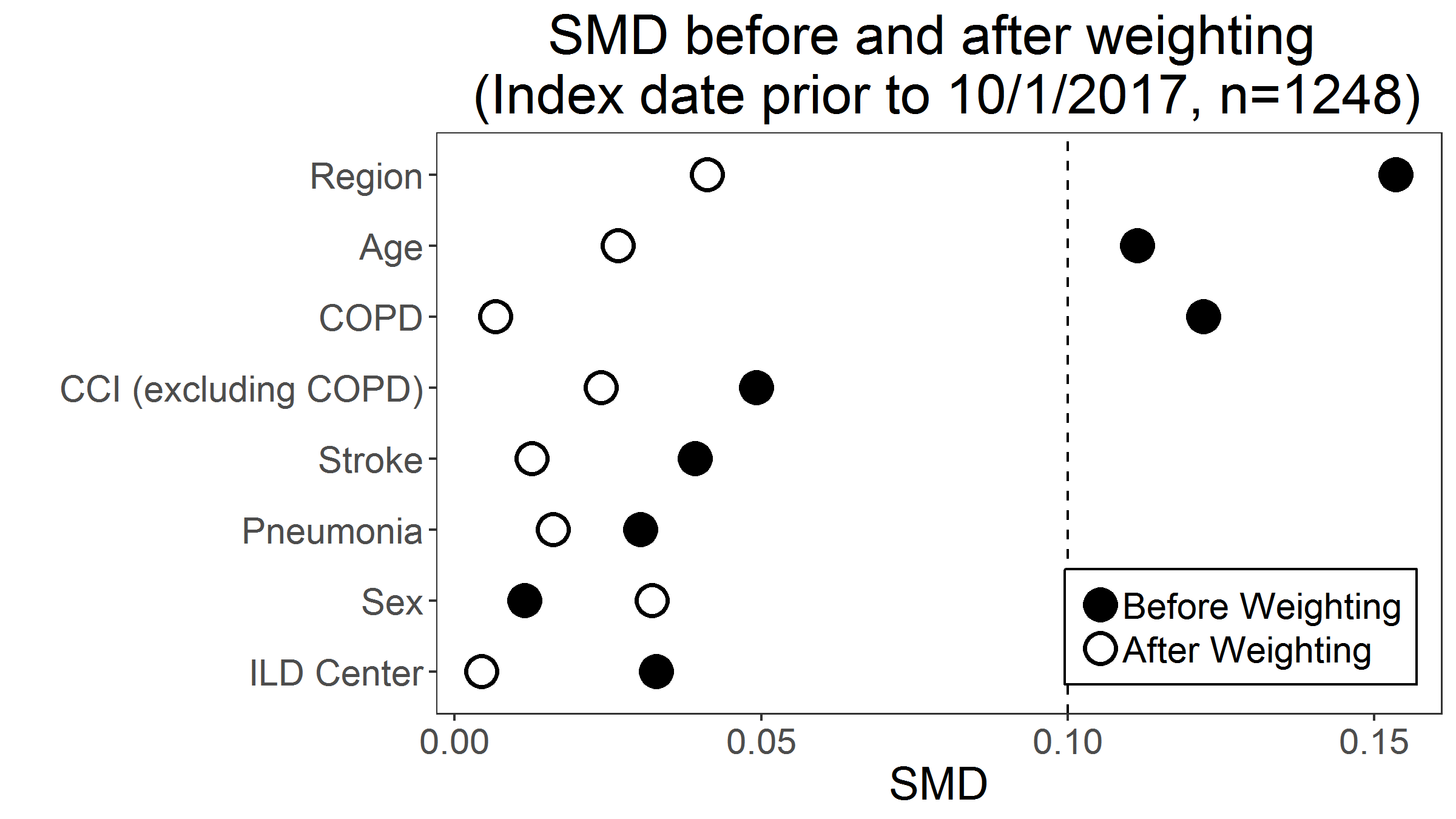


^1^Pirfenidone vs. nintedanib

^2^Pnemonia diagnosis in the three months immediately preceding the index date

^3^Interstitial Lung Disease (*ILD*) centers of excellence were defined as medical centers with specific expertise in the treatment of pulmonary fibrosis, as recognized by the Pulmonary Fibrosis Foundation.

CCI, Carlson Comorbidity Index; COPD, chronic obstructive pulmonary disease; SMD, standardized mean difference
